# A triclinic polymorph with *Z* = 3 of *N*,*N*′-bis­(2-pyrid­yl)oxamide

**DOI:** 10.1107/S1600536811010294

**Published:** 2011-03-26

**Authors:** Wan-Ju Zhang, Fang Wang, Gui-Ling Zhang, Xin Xiao

**Affiliations:** a School of Chemical Engineering, Huanggang Normal University, 438000 Huanggang, People’s Republic of China; b Experimental Center, Guiyang University, 550005 Guiyang, People’s Republic of China; c Institute of Applied Chemistry, Guizhou University, 550025 Guiyang, People’s Republic of China

## Abstract

The asymmetric unit of the title compound, C_12_H_10_N_4_O_2_, contains three half-mol­ecules. Each half-mol­ecule is completed by crystallographic inversion symmetry. The title compound, (I), is a polymorph of the structure, (II), reported by Hsu & Chen [*Eur. J. Inorg. Chem.* (2004), 1488–1493]. In the original report, the compound crystallized in the tetra­gonal space group *P* 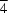
2_1_c (*Z* = 8), whereas the structure reported here is triclinic (*P* 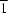, *Z* = 3). In both forms, each oxamide mol­ecule is almost planar (with maximum deviations are 0.266 and 0.166 Å) and the O atoms are *trans* oriented. The principal difference between the two forms lies in the different hydrogen-bonding patterns. In (I), two N—H⋯O and one N—H⋯N hydrogen bonds link the mol­ecules, forming a two-dimensional network, whereas in (II) there are no classical hydrogen bonds to O atoms and only weak C—H⋯O inter­actions are found along with rings of N—H⋯N bonds.

## Related literature

For general background to the use of *N*,*N*′-disubstituted oxamides as ligands, see: Bencini *et al.* (1986 ▶). For the synthesis and related structure, see: Hsu & Chen (2004 ▶).
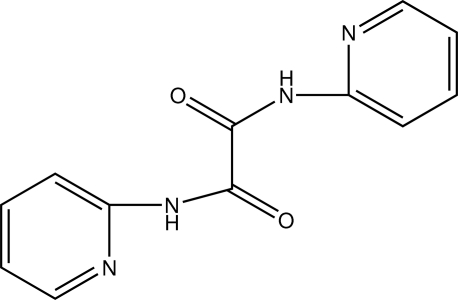


## Experimental

### 

#### Crystal data

C_12_H_10_N_4_O_2_
*M* *_r_* = 242.24
Triclinic, 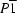

*a* = 8.459 (2) Å
*b* = 10.705 (3) Å
*c* = 11.058 (3) Å
α = 99.555 (9)°
β = 101.344 (8)°
γ = 112.980 (8)°
*V* = 870.5 (4) Å^3^
*Z* = 3
Mo *K*α radiation
μ = 0.10 mm^−1^
*T* = 298 K
0.26 × 0.26 × 0.20 mm

#### Data collection

Bruker SMART APEX CCD diffractometer
Absorption correction: multi-scan (*SADABS*; Sheldrick, 2003 ▶) *T*_min_ = 0.975, *T*_max_ = 0.980
8909 measured reflections
3018 independent reflections
2575 reflections with *I* > 2σ(*I*)
*R*_int_ = 0.021

#### Refinement

*R*[*F* ^2^ > 2σ(*F* ^2^)] = 0.044
*wR*(*F* ^2^) = 0.125
*S* = 1.07
3018 reflections
245 parameters
H-atom parameters constrained
Δρ_max_ = 0.24 e Å^−3^
Δρ_min_ = −0.34 e Å^−3^

### 

Data collection: *SMART* (Bruker, 2002 ▶); cell refinement: *SAINT* (Bruker, 2002 ▶); data reduction: *SAINT*; program(s) used to solve structure: *SHELXS97* (Sheldrick, 2008 ▶); program(s) used to refine structure: *SHELXL97* (Sheldrick, 2008 ▶); molecular graphics: *XP* (Siemens, 1994 ▶) and *CAMERON* (Watkin *et al.*, 1993 ▶); software used to prepare material for publication: *WinGX* (Farrugia, 1999 ▶).

## Supplementary Material

Crystal structure: contains datablocks global, I. DOI: 10.1107/S1600536811010294/ez2234sup1.cif

Structure factors: contains datablocks I. DOI: 10.1107/S1600536811010294/ez2234Isup2.hkl

Additional supplementary materials:  crystallographic information; 3D view; checkCIF report

## Figures and Tables

**Table 1:** Hydrogen-bond geometry (Å, °)

*D*—H⋯*A*	*D*—H	H⋯*A*	*D*⋯*A*	*D*—H⋯*A*
N2—H2*A*⋯O3	0.86	2.39	3.176 (2)	153
N4—H4*A*⋯O3	0.86	2.27	2.898 (2)	130
N6—H6⋯N3^i^	0.86	2.39	3.188 (2)	155

Symmetry code: (i) 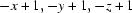.

**Fig. 1.**
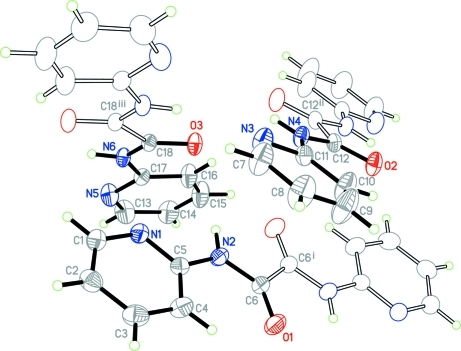
The molecular structure of (I), with atom labels and 30% probability displacement ellipsoids for non-H atoms. [symmetry codes: (i) -x + 1, -y, -z + 1; (ii) -x, -y, -z + 1; (iii) -x + 1, -y + 1, -z + 1.]

**Fig. 2.**
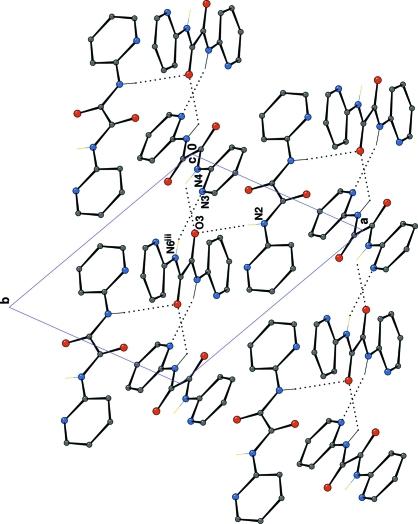
The two-dimensional hydrogen-bonded structure. Dashed lines indicate hydrogen bonds. H atoms not involved in hydrogen bonding were omitted for clarity. [symmetry code: (iii) -x + 1, -y + 1, -z + 1.]

## supplementary crystallographic information

### Comment

*N*,*N*'-disubstituted oxamides are known to be versatile organic ligands since their coordinating ability toward transition-metal ions can be modified and tuned by changing the nature of the amide substituents (Bencini *et al.*, 1986). The title compound, (I), is a triclinic polymorph of the previously reported crystal structure of this symmetrical N,*N*'-disubstituted oxamide which crystallizes in the tetragonal space group P-42_1_/c (Hsu & Chen, 2004). The relative arrangement of the molecules observed in the current structure is distinctively different from that previously reported.

The molecular structure of (I) is shown in Fig. 1. It crystallizes in the space group P-1 with three molecules in each unit cell. Each *N*,*N*'-di(2-pyridyl)oxamide molecule is almost planar and the O atoms are *trans*-oriented. In the crystal structure, classical N—H···O and N—H···N hydrogen bonds (Table 1, Fig. 2) link the molecules to form a two-dimensional network.

### Experimental

2-Aminopyridine (4.7 g, 50 mmol) was dissolved in 200 ml CH_2_Cl_2_, followed by addition of triethyl amine (10.0 ml, 72.1 mmol) at 273 K. The mixture was then stirred for 10 min. Oxalyl chloride (2.2 ml, 25 mmol) in 10 ml CH_2_Cl_2_ was then added slowly to the above mixture. After continuous stirring for about 3 h at 273 K, the resulting solution was concentrated under vacuum until a large amount of solid precipitated. The solid was filtered, washed with water and then dried in vacuum. Yield: 4.3 g (71%). Colourless block crystals suitable for X-ray crystallography were obtained by slow evaporation of the solvent from a solution of the title compound in toluene.

### Refinement

All the hydrogen atoms were placed in calculated positions, with C—H distances of 0.93 Å (aromatic) and N—H distance of 0.86 Å, and were included in the final cycles of refinement as riding, with *U*_iso_(H) = 1.2*U*_eq_ of the carrier atom.

### Crystal data

**Table:**

C_12_H_10_N_4_O_2_	*Z* = 3
*M**_r_* = 242.24	*F*(000) = 378
Triclinic, *P*1	*D*_x_ = 1.386 Mg m^−^^3^
Hall symbol: -p 1	Mo *K*α radiation, λ = 0.71073 Å
*a* = 8.459 (2) Å	Cell parameters from 2575 reflections
*b* = 10.705 (3) Å	θ = 2.0–25.1°
*c* = 11.058 (3) Å	µ = 0.10 mm^−^^1^
α = 99.555 (9)°	*T* = 298 K
β = 101.344 (8)°	Block, colourless
γ = 112.980 (8)°	0.26 × 0.26 × 0.20 mm
*V* = 870.5 (4) Å^3^	

### Data collection

**Table:**

Bruker SMART APEX CCD diffractometer	3018 independent reflections
Radiation source: fine-focus sealed tube	2575 reflections with *I* > 2σ(*I*)
graphite	*R*_int_ = 0.021
φ and ω scans	θ_max_ = 25.1°, θ_min_ = 2.0°
Absorption correction: multi-scan (*SADABS*; Sheldrick, 2003)	*h* = −10→9
*T*_min_ = 0.975, *T*_max_ = 0.980	*k* = −12→12
8909 measured reflections	*l* = −13→13

### Refinement

**Table:**

Refinement on *F*^2^	Primary atom site location: structure-invariant direct methods
Least-squares matrix: full	Secondary atom site location: difference Fourier map
*R*[*F*^2^ > 2σ(*F*^2^)] = 0.044	Hydrogen site location: inferred from neighbouring sites
*wR*(*F*^2^) = 0.125	H-atom parameters constrained
*S* = 1.07	*w* = 1/[σ^2^(*F*_o_^2^) + (0.0745*P*)^2^ + 0.1649*P*] where *P* = (*F*_o_^2^ + 2*F*_c_^2^)/3
3018 reflections	(Δ/σ)_max_ < 0.001
245 parameters	Δρ_max_ = 0.24 e Å^−^^3^
0 restraints	Δρ_min_ = −0.34 e Å^−^^3^

### Special details

**Table:**

Geometry. All e.s.d.'s (except the e.s.d. in the dihedral angle between two l.s. planes) are estimated using the full covariance matrix. The cell e.s.d.'s are taken into account individually in the estimation of e.s.d.'s in distances, angles and torsion angles; correlations between e.s.d.'s in cell parameters are only used when they are defined by crystal symmetry. An approximate (isotropic) treatment of cell e.s.d.'s is used for estimating e.s.d.'s involving l.s. planes.
Refinement. Refinement of *F*^2^ against ALL reflections. The weighted *R*-factor *wR* and goodness of fit *S* are based on *F*^2^, conventional *R*-factors *R* are based on *F*, with *F* set to zero for negative *F*^2^. The threshold expression of *F*^2^ > σ(*F*^2^) is used only for calculating *R*-factors(gt) *etc*. and is not relevant to the choice of reflections for refinement. *R*-factors based on *F*^2^ are statistically about twice as large as those based on *F*, and *R*- factors based on ALL data will be even larger.

### Fractional atomic coordinates and isotropic or equivalent isotropic displacement parameters (Å^2^)

**Table:**

	*x*	*y*	*z*	*U*_iso_*/*U*_eq_	
C1	0.8362 (2)	0.46853 (19)	0.37248 (18)	0.0520 (4)	
H1	0.8499	0.5608	0.3908	0.062*	
C2	0.9305 (2)	0.4331 (2)	0.29625 (17)	0.0530 (5)	
H2	1.0085	0.5002	0.2655	0.064*	
C3	0.9066 (3)	0.2958 (2)	0.26675 (17)	0.0557 (5)	
H3	0.9672	0.2682	0.2144	0.067*	
C4	0.7921 (3)	0.1992 (2)	0.31520 (17)	0.0525 (4)	
H4	0.7732	0.1056	0.2957	0.063*	
C5	0.7062 (2)	0.24577 (17)	0.39383 (15)	0.0413 (4)	
C6	0.5610 (2)	0.02730 (17)	0.45799 (16)	0.0433 (4)	
C7	0.2643 (3)	0.1234 (2)	0.11856 (19)	0.0686 (6)	
H7	0.3133	0.2017	0.0881	0.082*	
C8	0.2515 (4)	−0.0019 (2)	0.0534 (2)	0.0805 (7)	
H8	0.2911	−0.0087	−0.0191	0.097*	
C9	0.1791 (4)	−0.1164 (2)	0.0977 (2)	0.0880 (8)	
H9	0.1681	−0.2033	0.0552	0.106*	
C10	0.1222 (3)	−0.1038 (2)	0.20524 (19)	0.0662 (6)	
H10	0.0731	−0.1809	0.2371	0.079*	
C11	0.1405 (2)	0.02693 (16)	0.26431 (14)	0.0386 (4)	
C12	0.0128 (2)	−0.03698 (15)	0.43944 (14)	0.0364 (3)	
C13	0.7504 (3)	0.5115 (2)	0.97931 (17)	0.0646 (6)	
H13	0.8479	0.5692	1.0504	0.078*	
C14	0.6308 (3)	0.3874 (2)	0.99039 (17)	0.0631 (5)	
H14	0.6460	0.3612	1.0664	0.076*	
C15	0.4884 (3)	0.3032 (2)	0.88626 (18)	0.0660 (6)	
H15	0.4042	0.2175	0.8901	0.079*	
C16	0.4690 (3)	0.34467 (18)	0.77573 (16)	0.0521 (5)	
H16	0.3719	0.2886	0.7040	0.063*	
C17	0.5974 (2)	0.47196 (15)	0.77397 (13)	0.0347 (3)	
C18	0.48440 (19)	0.45519 (14)	0.54712 (13)	0.0312 (3)	
N1	0.72567 (19)	0.37762 (14)	0.42180 (14)	0.0472 (4)	
N2	0.59256 (19)	0.15935 (14)	0.45411 (14)	0.0457 (4)	
H2A	0.5362	0.1959	0.4934	0.055*	
N3	0.2108 (2)	0.14008 (15)	0.22304 (13)	0.0505 (4)	
N4	0.08990 (17)	0.05422 (13)	0.37520 (12)	0.0385 (3)	
H4A	0.1109	0.1402	0.4060	0.046*	
N5	0.7374 (2)	0.55612 (16)	0.87343 (13)	0.0531 (4)	
N6	0.59098 (16)	0.52565 (12)	0.66636 (11)	0.0351 (3)	
H6	0.6628	0.6127	0.6784	0.042*	
O1	0.62022 (18)	−0.04585 (13)	0.40434 (13)	0.0584 (4)	
O2	−0.03443 (16)	−0.16334 (11)	0.41046 (11)	0.0501 (3)	
O3	0.37249 (15)	0.33253 (10)	0.51223 (9)	0.0415 (3)	

### Atomic displacement parameters (Å^2^)

**Table:**

	*U*^11^	*U*^22^	*U*^33^	*U*^12^	*U*^13^	*U*^23^
C1	0.0499 (10)	0.0450 (10)	0.0644 (11)	0.0197 (8)	0.0202 (9)	0.0213 (8)
C2	0.0461 (10)	0.0640 (12)	0.0538 (10)	0.0229 (9)	0.0177 (8)	0.0273 (9)
C3	0.0585 (11)	0.0729 (13)	0.0507 (10)	0.0377 (10)	0.0235 (9)	0.0228 (9)
C4	0.0628 (11)	0.0524 (10)	0.0545 (10)	0.0336 (9)	0.0223 (9)	0.0177 (8)
C5	0.0377 (8)	0.0414 (9)	0.0461 (9)	0.0190 (7)	0.0099 (7)	0.0139 (7)
C6	0.0408 (9)	0.0362 (8)	0.0523 (9)	0.0183 (7)	0.0095 (7)	0.0116 (7)
C7	0.1028 (17)	0.0534 (11)	0.0552 (11)	0.0248 (11)	0.0486 (12)	0.0216 (9)
C8	0.123 (2)	0.0638 (13)	0.0675 (13)	0.0354 (13)	0.0651 (14)	0.0193 (11)
C9	0.144 (2)	0.0524 (12)	0.0840 (16)	0.0407 (14)	0.0736 (17)	0.0163 (11)
C10	0.1025 (16)	0.0397 (9)	0.0627 (12)	0.0248 (10)	0.0487 (12)	0.0162 (9)
C11	0.0413 (8)	0.0363 (8)	0.0350 (8)	0.0117 (7)	0.0144 (6)	0.0106 (6)
C12	0.0348 (8)	0.0306 (8)	0.0382 (8)	0.0075 (6)	0.0119 (6)	0.0110 (6)
C13	0.0660 (12)	0.0720 (13)	0.0381 (9)	0.0167 (10)	0.0012 (8)	0.0205 (9)
C14	0.0823 (14)	0.0694 (13)	0.0403 (10)	0.0300 (11)	0.0168 (9)	0.0304 (9)
C15	0.0864 (15)	0.0509 (11)	0.0469 (11)	0.0109 (10)	0.0205 (10)	0.0253 (9)
C16	0.0639 (11)	0.0433 (9)	0.0350 (8)	0.0078 (8)	0.0133 (8)	0.0149 (7)
C17	0.0420 (8)	0.0353 (8)	0.0308 (7)	0.0176 (7)	0.0145 (6)	0.0123 (6)
C18	0.0351 (7)	0.0296 (7)	0.0309 (7)	0.0125 (6)	0.0151 (6)	0.0104 (6)
N1	0.0447 (8)	0.0396 (8)	0.0619 (9)	0.0187 (6)	0.0209 (7)	0.0175 (7)
N2	0.0473 (8)	0.0368 (7)	0.0613 (9)	0.0224 (6)	0.0219 (7)	0.0164 (6)
N3	0.0719 (10)	0.0409 (8)	0.0412 (7)	0.0186 (7)	0.0299 (7)	0.0159 (6)
N4	0.0484 (8)	0.0292 (6)	0.0365 (7)	0.0121 (6)	0.0186 (6)	0.0102 (5)
N5	0.0540 (9)	0.0531 (9)	0.0366 (7)	0.0098 (7)	0.0052 (6)	0.0164 (6)
N6	0.0408 (7)	0.0291 (6)	0.0311 (6)	0.0088 (5)	0.0122 (5)	0.0112 (5)
O1	0.0672 (8)	0.0431 (7)	0.0791 (9)	0.0310 (6)	0.0337 (7)	0.0199 (6)
O2	0.0628 (8)	0.0309 (6)	0.0526 (7)	0.0104 (5)	0.0290 (6)	0.0117 (5)
O3	0.0489 (6)	0.0306 (6)	0.0343 (6)	0.0053 (5)	0.0129 (5)	0.0108 (4)

### Geometric parameters (Å, °)

**Table:**

C1—N1	1.336 (2)	C11—N3	1.326 (2)
C1—C2	1.373 (3)	C11—N4	1.399 (2)
C1—H1	0.9300	C12—O2	1.2149 (18)
C2—C3	1.375 (3)	C12—N4	1.3411 (18)
C2—H2	0.9300	C12—C12^ii^	1.537 (3)
C3—C4	1.378 (3)	C13—N5	1.337 (2)
C3—H3	0.9300	C13—C14	1.363 (3)
C4—C5	1.386 (2)	C13—H13	0.9300
C4—H4	0.9300	C14—C15	1.361 (3)
C5—N1	1.330 (2)	C14—H14	0.9300
C5—N2	1.410 (2)	C15—C16	1.370 (2)
C6—O1	1.219 (2)	C15—H15	0.9300
C6—N2	1.342 (2)	C16—C17	1.378 (2)
C6—C6^i^	1.535 (3)	C16—H16	0.9300
C7—N3	1.331 (2)	C17—N5	1.323 (2)
C7—C8	1.365 (3)	C17—N6	1.4054 (18)
C7—H7	0.9300	C18—O3	1.2193 (18)
C8—C9	1.360 (3)	C18—N6	1.3384 (19)
C8—H8	0.9300	C18—C18^iii^	1.523 (3)
C9—C10	1.374 (3)	N2—H2A	0.8600
C9—H9	0.9300	N4—H4A	0.8600
C10—C11	1.377 (2)	N6—H6	0.8600
C10—H10	0.9300		
			
N1—C1—C2	123.58 (17)	O2—C12—N4	126.93 (14)
N1—C1—H1	118.2	O2—C12—C12^ii^	121.30 (15)
C2—C1—H1	118.2	N4—C12—C12^ii^	111.77 (15)
C1—C2—C3	118.10 (17)	N5—C13—C14	124.56 (18)
C1—C2—H2	121.0	N5—C13—H13	117.7
C3—C2—H2	121.0	C14—C13—H13	117.7
C2—C3—C4	119.68 (17)	C15—C14—C13	117.60 (16)
C2—C3—H3	120.2	C15—C14—H14	121.2
C4—C3—H3	120.2	C13—C14—H14	121.2
C3—C4—C5	117.98 (17)	C14—C15—C16	119.96 (17)
C3—C4—H4	121.0	C14—C15—H15	120.0
C5—C4—H4	121.0	C16—C15—H15	120.0
N1—C5—C4	123.16 (15)	C15—C16—C17	118.07 (17)
N1—C5—N2	113.25 (14)	C15—C16—H16	121.0
C4—C5—N2	123.58 (15)	C17—C16—H16	121.0
O1—C6—N2	126.53 (16)	N5—C17—C16	123.44 (14)
O1—C6—C6^i^	120.90 (18)	N5—C17—N6	113.46 (13)
N2—C6—C6^i^	112.56 (18)	C16—C17—N6	123.09 (14)
N3—C7—C8	123.90 (18)	O3—C18—N6	126.12 (12)
N3—C7—H7	118.1	O3—C18—C18^iii^	121.04 (16)
C8—C7—H7	118.1	N6—C18—C18^iii^	112.84 (15)
C9—C8—C7	117.97 (19)	C5—N1—C1	117.47 (15)
C9—C8—H8	121.0	C6—N2—C5	128.27 (15)
C7—C8—H8	121.0	C6—N2—H2A	115.9
C8—C9—C10	120.0 (2)	C5—N2—H2A	115.9
C8—C9—H9	120.0	C11—N3—C7	117.05 (15)
C10—C9—H9	120.0	C12—N4—C11	128.10 (13)
C9—C10—C11	117.76 (17)	C12—N4—H4A	115.9
C9—C10—H10	121.1	C11—N4—H4A	115.9
C11—C10—H10	121.1	C17—N5—C13	116.37 (15)
N3—C11—C10	123.32 (15)	C18—N6—C17	126.63 (12)
N3—C11—N4	113.26 (14)	C18—N6—H6	116.7
C10—C11—N4	123.41 (14)	C17—N6—H6	116.7

Symmetry codes: (i) −*x*+1, −*y*, −*z*+1; (ii) −*x*, −*y*, −*z*+1; (iii) −*x*+1, −*y*+1, −*z*+1.

### Hydrogen-bond geometry (Å, °)

**Table:**

*D*—H···*A*	*D*—H	H···*A*	*D*···*A*	*D*—H···*A*
N2—H2A···O3	0.86	2.39	3.176 (2)	153
N4—H4A···O3	0.86	2.27	2.898 (2)	130
N6—H6···N3^iii^	0.86	2.39	3.188 (2)	155

Symmetry codes: (iii) −*x*+1, −*y*+1, −*z*+1.

## References

[bb1] Bencini, A., Benelli, C., Fabretti, A. C., Franchini, G. & Gatteschi, D. (1986). *Inorg. Chem.* **25**, 1063–1066.

[bb2] Bruker (2002). *SAINT* and *SMART* Bruker AXS Inc., Madison, Wisconsin, USA.

[bb3] Farrugia, L. J. (1999). *J. Appl. Cryst.* **32**, 837–838.

[bb4] Hsu, Y.-F. & Chen, J.-D. (2004). *Eur. J. Inorg. Chem.* pp. 1488–1493.

[bb5] Sheldrick, G. M. (2003). *SADABS* University of Göttingen, Germany.

[bb6] Sheldrick, G. M. (2008). *Acta Cryst.* A**64**, 112–122. 10.1107/S0108767307043930 18156677

[bb7] Siemens (1994). *XP* Siemens Analytical X-ray Instruments Inc., Madison, Wisconsin, USA.

[bb8] Watkin, D. M., Pearce, L. & Prout, C. K. (1993). *CAMERON* Chemical Crystallography Laboratory, University of Oxford, England.

